# Genome Sequence of Carbapenemase-Producing Enterobacter cloacae 0102-4P-1 Harboring the IncC-Type Plasmid with a Multidrug Resistance Site Encoding *bla*_NDM-1_, Isolated from Commercially Imported Shrimp

**DOI:** 10.1128/mra.01058-21

**Published:** 2022-04-20

**Authors:** Tatsuya Nakayama, Takahiro Yamaguchi, Shiori Yamamoto, Michio Jinnai, Yuko Kumeda, Atsushi Hase

**Affiliations:** a Graduate School of Integrated Sciences for Life, Hiroshima University, Hiroshima, Japan; b Division of Biomedical Food Research, National Institute of Health Sciences, Kawasaki, Japan; c Division of Microbiology, Osaka Institute of Public Health, Osaka, Japan; d Department of Microbiology, Kanagawa Prefectural Institute of Public Health, Kanagawa, Japan; e Research Center for Microorganism Control, Osaka Prefecture University, Osaka, Japan; f Faculty of Contemporary Human Life Science, Tezukayama University, Nara, Japan; University of Maryland School of Medicine

## Abstract

A carbapenem-resistant Enterobacter cloacae 0102-4P-1 strain was isolated from commercially imported shrimp in Japan. Here, we present a draft genome sequence. The complete plasmid sequence was also determined by hybrid assembly sequencing using Oxford Nanopore and Illumina methods. The assembled whole genome and plasmid were 5,164,033 bp and 162,852 bp long, respectively.

## ANNOUNCEMENT

Antibiotic-resistant bacteria that contaminate food can be transferred to humans (1). Research interest in the treatment of life-threatening infections caused by carbapenem-resistant bacteria (1), which typically possess plasmid-mediated antibiotic resistance (2), is increasing. The spread of antibiotic resistance through plasmid-mediated horizontal gene transfer is an important aspect of this study.

We previously isolated carbapenem-resistant Enterobacter cloacae 0102-4P-1 from shrimp imported to Japan from Vietnam (3). Here, we describe a draft whole-genome sequence (WGS) and complete plasmid sequence (PS) of E. cloacae 0102-4P-1. In addition, a plasmid map harboring *bla*_NDM-1_ was obtained and compared with maps of highly homologous plasmids.

E. cloacae 0102-4P-1 was incubated at 37°C for 18 h in tryptic soy agar (Becton, Dickinson, Franklin Lakes, NJ). DNA was extracted using the DNeasy blood and tissue kit (Qiagen, Hilden, Germany) for short reads and Genomic-tip 100/G (Qiagen) for long reads. Short- and long-read sequencing libraries were prepared using Nextera DNA flex library kit (Illumina, San Diego, CA) and the rapid barcoding kit (SQK-RBK004; Oxford Nanopore Technologies, Oxford, UK), respectively. Short- and long-read sequencing were performed using the Illumina iSeq100 system with a 2 × 150-bp paired-end protocol and Oxford Nanopore MinION device with flow cell R9.4.1 (FLO-MIN106D) and MinKNOW software v20.10.3. The obtained fast5 reads were based on Guppy v4.2.3. Short-read sequences were trimmed and quality checks performed using fastp v0.20.0 (4). Short- and long-read quality checks were performed using fastqc v0.11.9 and NanoPlot v1.38.0 (5). A hybrid assembly of Illumina (total reads, 17,136,820; mean length after filtering, 2 × 143-bp reads [paired end]; total bases, 246.5 Mb; coverage, 51×) and MinION (total reads, 48,182; *N*_50_ value, 5,335 bp; total bases, 171.9 Mb) sequencing data was performed using Unicycler v0.4.8 (6). The Unicycler bridge assembly graph determined that the contig 3 was completely cyclic (pEC0102-4P-1). Default parameters were used except where otherwise noted. Annotation was performed using DFAST software. The assembled WGS was analyzed for the E. cloacae complex using SpeciesFinder2.0, MLST2.0, and ResFinder4.1 (Table 1).

**TABLE 1:** Genome information for E. cloacae 0102-4P-1 and pEC0102-4P-01

Parameter	Data for:	
	E. cloacae 0102-4P-1	pEC0102-4P-01
Sequencing type	Whole genome	Plasmid
Total genome size (bp)	5,164,033	162,852
No. of sequences	13	1
GC content (%)	54.8	52.2
No. of coding DNA sequences	4,920	198
No. of rRNAs	21	0
No. of tRNAs	83	0
Coding ratio (%)	88.8	87.8
Genotype^*a*^	Multilocus sequence typing, unknown; *dnaA*: 43, *fusA*:24, *gyrB*:66, *leuS*:247, *pyrG*:3, *rplB*:15, *rpoB*:3	Plasmid Inc-type, IncC
Accession no.	BPMY01000001	AP024844
Antibiotic resistance genes	*dfrA16*, *aadA2b*, *sul1*, *bla*_NDM-1_	
	*fosA*, *bla*_ACT-9_	

a Multi-locus sequence typing allele profile.

**FIG 1:**
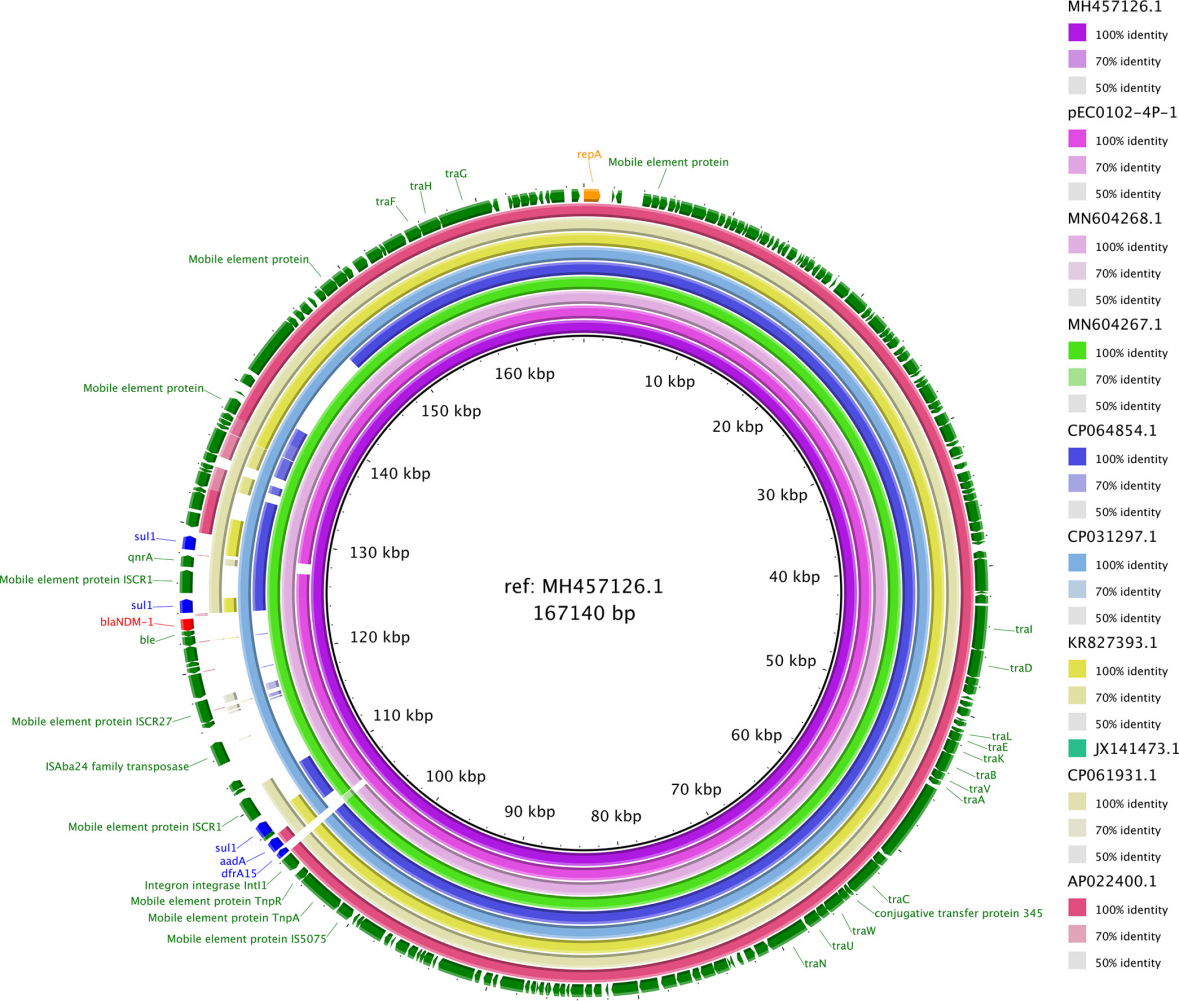
Complete plasmid map of pEC0102-4P-01 from E. cloacae 0102-4P-1. The complete genomes of nine strains (GenBank accession no. MH457126, MN604268, MN604267, CP031297, CP064854, KR827393, JX141473, CP061931, and AP022400) with high homology (99.97% to 99.99%) were selected. The reference strain was MH457126. Plasmid maps were designed using BLAST Ring Image Generator v0.95. The plasmid map showed that four plasmids (MH457126, MN604268, MN604267, and CP031297) had approximately 20- to 30-kbp multidrug regions encoding antibiotic-resistance genes (ARGs), including *bla*_NDM-1_, mobile element proteins, and IS*Aba24* family transposase.

S1 nuclease pulsed-field gel electrophoresis and Southern hybridization were performed to confirm the localization of *bla*_NDM-1_ (7). WGS analysis showed that contig 3 encoding *bla*_NDM_ was the same size as the plasmid encoding *bla*_NDM-1_. Hence, contig 3 was analyzed using PlasmidFinder2.1. BLAST was used to determine the PS similarity of pEC0102-4P-1. The E. cloacae WGS is 5,164,033 bp (Table 1). PS contig 3 (pEC0102-4P-1) is 162,852 bp. Plasmid typing was performed using an Inc-type plasmid (Table 1). To clarify the plasmid encoding *bla*_NDM-1_, a complete map was designed, and the localization of *bla*_NDM-1_ was compared with that of a high homology plasmid (Fig. 1). Based on the composition of these genes, it is conceivable that the multidrug resistance site could spread horizontally to other plasmids. There have been no reports of carbapenem-resistant E. cloacae with an IncC-type plasmid harboring *bla*_NDM-1_ in seafood. Data from plasmid sequencing studies, such as ours, may reveal more information regarding plasmids encoding carbapenemase-related genes in seafood.

### Data availability.

The draft WGS of E. cloacae 0102-4P-1 and PS of pEC0102-4P-1 from E. cloacae 0102-4P-1 were deposited in DDBJ/GenBank (accession numbers BPMY01000001 and AP024844). The raw reads were deposited under accession numbers DRX313430 and DRX313429 with BioProject number PRJDB11927.
